# Supplementary material for: Aftershock sequences and seismic-like organization of acoustic events produced by a single propagating crack
Source: Nat Commun. 2018 Mar 28;9:1253. doi: 10.1038/s41467-018-03559-4 (PMC5871842; doi:10.1038/s41467-018-03559-4)
Supplement: Supplementary file 1 — Supplementary Information [file 41467_2018_3559_MOESM1_ESM.pdf]

Supplementary Information for "Aftershock sequences and seismic-like organization of acoustic events produced by a single propagating crack"

Jonathan Barés[1,2], Alizée Dubois[1], Lamine Hattali[1,3], Davy Dalmas[4] & Daniel Bonamy[1,*]

[1]*Service de Physique de l'Etat Condensé, CEA, CNRS, Université Paris-Saclay, CEA Saclay 91191 Gif-sur-Yvette Cedex, France*

[2]*Laboratoire de Mécanique et Génie Civil, Université de Montpellier, CNRS, 163 rue Auguste Broussonnet, 34090 Montpellier, France.*

[3]*Laboratoire FAST, Univ. Paris-Sud, CNRS, Université Paris-Saclay, F-91405, Orsay, France.*

[4]*Laboratoire de Tribologie et Dynamique des Systemes, CNRS, Ecole Centrale de Lyon, 36, Avenue Guy de Collongue, 69134 Ecully Cedex, France.*

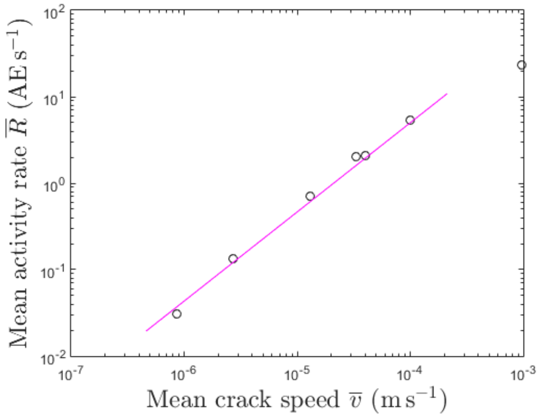

Supplementary Figure 1: **Mean activity rate, $\overline{R}$, as a function of the mean crack speed,** $\overline{v}$ over the considered time window. The axes are logarithmic. Here, the microstructure length-scale is $d = 583\,\mu\mathrm{m}$. Straight magenta line is a linear fit $\overline{R} = C\overline{v}$ with a fitted value $C = 45{\pm}14\,\mathrm{AE\,mm^{-1}}$.

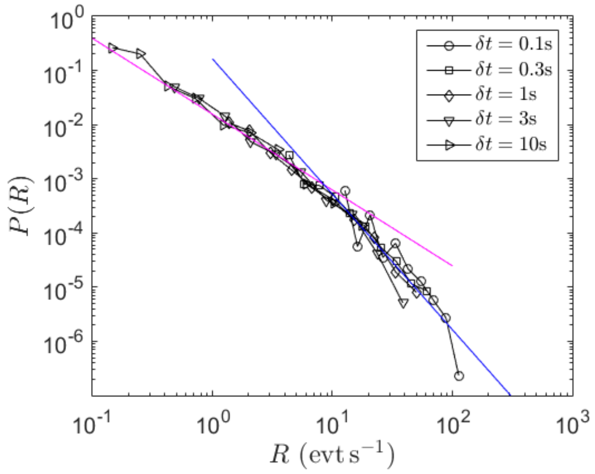

Supplementary Figure 2: **Distribution of instantaneous activity rate**. Note that, in experiments, an "instantaneous" quantity is actually averaged over a finite time scale δt whose value affects the fluctuation amplitude. The distribution of $R(t)$ was therefore computed for different δt (values indicated in the legend). The figure shows that all curves collapse onto a single master curve, exhibiting two power-law regimes: A small scale regime with a scaling exponent $a_{\text{small}} = 1.48 \pm 0.16$ (Leftward, magenta) and a large scale regime with a scaling exponent $a_{\text{large}} = 2.5 \pm 0.3$ (Rightward, blue). Since the mean activity rate is proportional to the mean crack speed (Supplementary Fig. 1), it is interesting to compare the above distributions with the distributions of the "instantaneous" crack speed, $v(t)$ (where the "instantaneous" velocity is defined over the same time scales δt). These have been analyzed in an earlier work [1]. It has been shown [1] that, as for $R(t)$, the distributions of $v(t)$ collapse onto a single master curve independent of δt, which exhibits two power-law regimes with similar exponents: $a_{\text{small}} = 1.4 \pm 0.15$ and $a_{\text{large}} = 2.5 \pm 0.1$. This suggests that the statement "mean activity rate proportional to mean crack speed" remains true even when the averaging process is performed over a finite (and relatively small) time scale δt. In this experiment, the microstructure length-scale is $d = 583\,\mu\text{m}$ and the crack speed $\overline{v} = 2.7\ \mu\text{m}\,\text{s}^{-1}$. The axes are logarithmic.

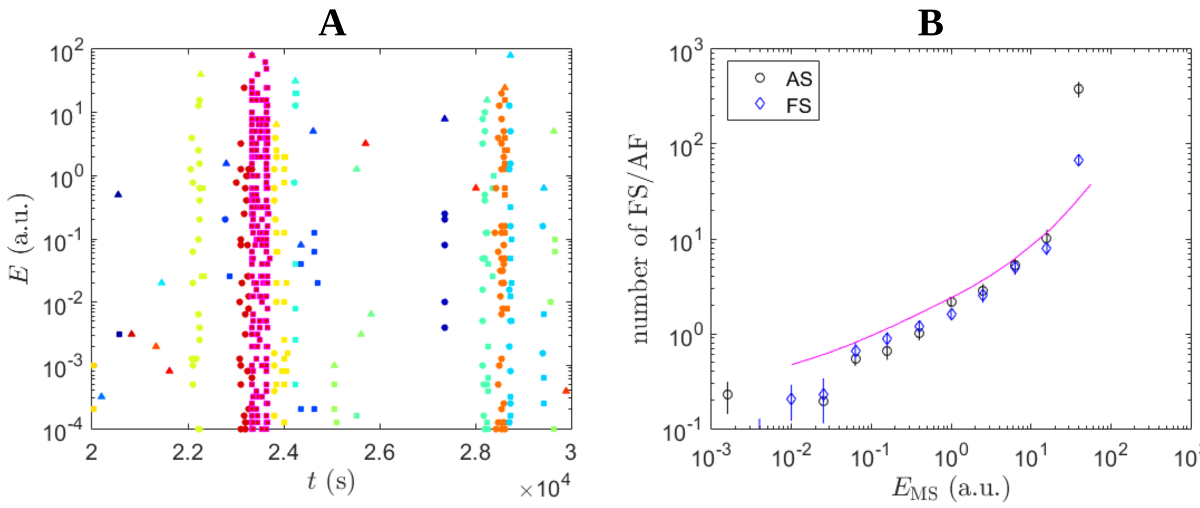

Supplementary Figure 3: **Sequence declustering based on inter-event times and so-obtained productivity law**. In seismology, the procedures used to cluster the earthquakes into sequences of dependent (AS) earthquakes are either based on the spatio-temporal proximity between the events and/or on the fact that the events within a same cluster occur at rates different from what is expected for a Poisson process. The former cannot be applied in our experiments due to the lack of spatial information of the AE sources. The latter is applied using a thinning procedure proposed by van Stiphout et al. [2] based on the work of Hainzl et al. [3]: (i) for each inter-event time Δt, we compute the probability $Prob = P(\Delta t)/BP_0(\Delta t)$ that Δt is a 'normal' value for two consecutive events in a Poisson process – here $P(\Delta t)$ is the measured pdf (given by Supplementary Eq. 1 with $u = \Delta t/\overline{R}$), $P_0(\Delta t) = (\overline{R}/B)\exp(-\overline{R}\Delta t/B)$ is the pdf for background events, and $1/B$ is the background fraction; (ii) we draw a random number x uniformly distributed between 0 and 1; (iii) if $x < Prob$ the two successive events are stated to belong to distinct sequences. **(a)** Typical zoomed view on the energy *vs.* time of occurrence of AE after declustering. y axis is logarithmic. Different colors correspond to different sequences. Symbols $\triangle$, o and $\square$ correspond to MS (event with the largest energy in the sequence), FS (events before MS) and AS (events after MS). **(b)** Mean number of AS (black o) and FS (blue $\square$), N_{AS} and N_{FS}, as a function of the MS energy E_{MS}. Errorbars indicate a 95% confident interval. Magenta line is the same curve as that in Fig. 3a (main text), predicted by Eq. 3 (main text), which was shown to fit extremely well the curve $N_{\mathrm{AS}}(E_{\mathrm{MS}})$ obtained with the declustering procedure proposed in the main text.

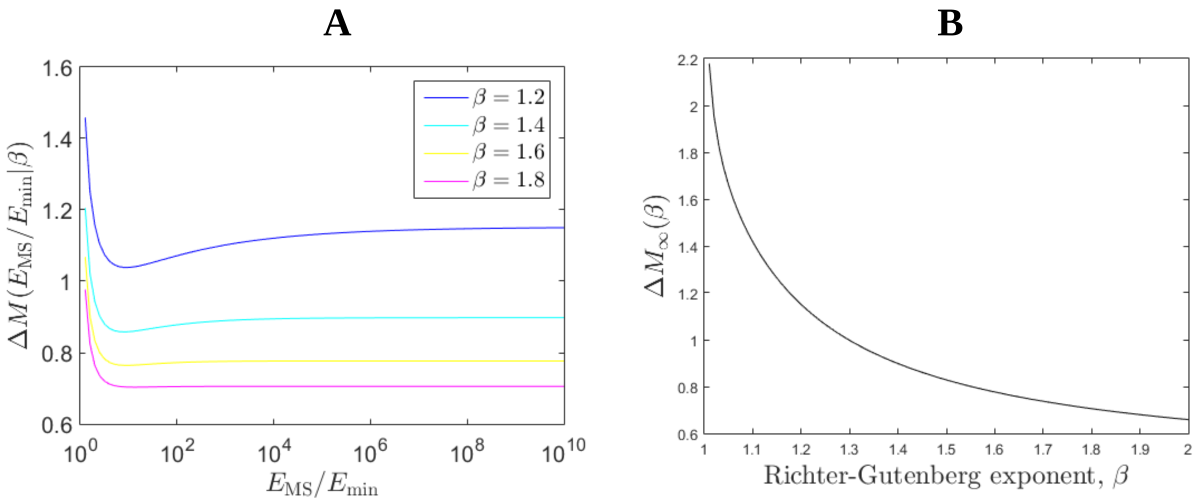

Supplementary Figure 4: **Bath law when the energy distribution takes a simple power-law form** $P(E) \propto E^{-\beta}$. **(a)** Difference in magnitude ΔM (here $M = \log_{10} E$) between the MS and its largest AS as a function of $E_{\rm MS}/E_{\rm min}$ for different values of the Gutenberg-Richter exponent β, as predicted by Supplementary Eq. 12. **(b)** Variation of asymptotic value $\Delta M(E_{\rm MS}/E_{\rm min} \to \infty|\beta)$ as a function of β.

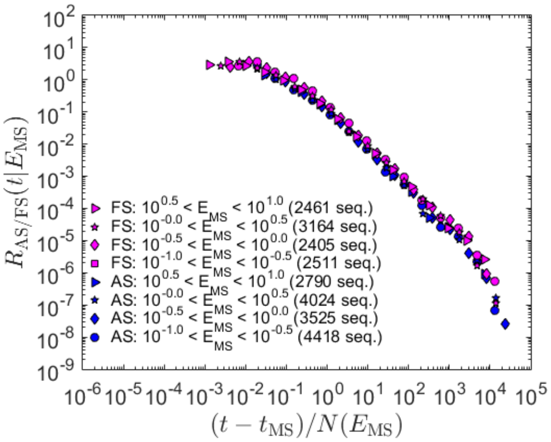

Supplementary Figure 5: **Stationary dynamics and symmetry between FS and AS production rate**. Blue symbols: Number of AS per unit time, $R_{\mathrm{AS}}(t|E_{\mathrm{MS}})$, as a function of elapsed time since the MS occurrence, $t - t_{\mathrm{MS}}$. Magenta symbols: Number of FS per unit time, $R_{\mathrm{FS}}(t|E_{\mathrm{MS}})$, as a function of remaining time to the MS occurrence, $t_{\mathrm{MS}} - t$. In both cases, the sequences have been sorted according to the MS energy indicated by the legend. Note the overlapping between the two.

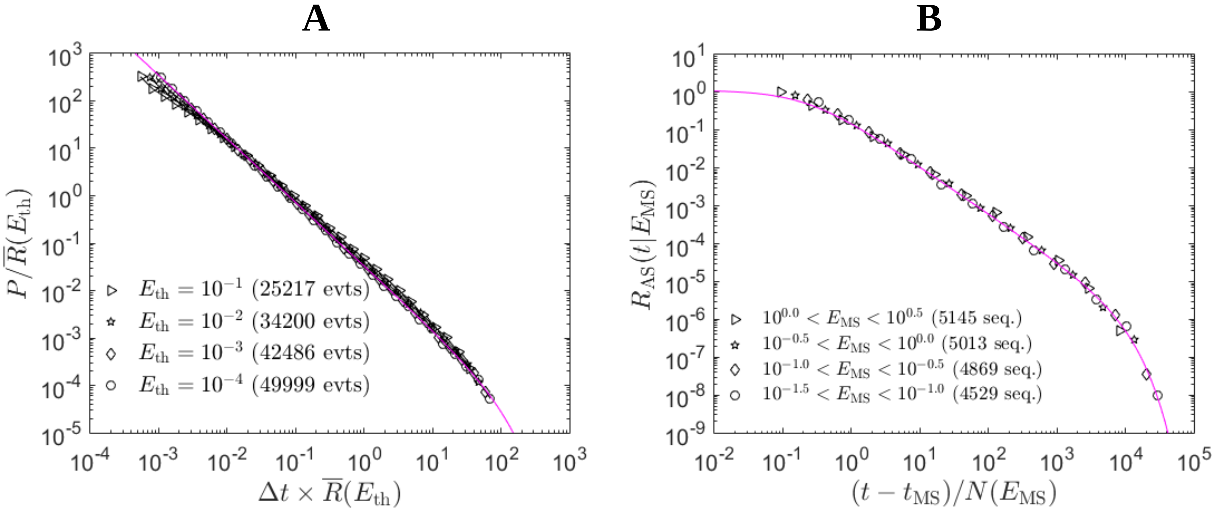

Supplementary Figure 6: **Scaled Omori-Utsu law in AS sequences as a consequence of the self-similar organization of events**. An artificial series $\{t, E\}$ was created so that E obeys the Gutenberg-Richter distribution (Main text Eq. 1) with $\{\beta = 0.96, E_{\rm min} = 10^{-4}, E_0 = 38\}$, and Δt obeys the gamma distribution $P(\Delta t) \propto \Delta t^{-\gamma} \exp(-\Delta t/\Delta t_0)$ for $\Delta t \geq \Delta t_{\rm min}$ with $\{\gamma = 1.34, \Delta t_{\rm min} = 7 \times 10^{-2}, \Delta t_0 = 10^4\}$. These parameters were chosen to be close to those measured in the experiment detailed in Fig. 2 main text. **(a)** Distribution of the scaled recurrence time $R(E_{\rm th}) \times \Delta t$ in the so-obtained artificial catalog. As in Fig. 2b main text, $R(E_{\rm th})$ denotes the mean activity rate for events with energy $E > E_{\rm th}$. The magenta curve is the gamma function $f(x) \propto x^{-\gamma} \exp(-x/B)$ for $x \geq b$ with fitted parameters $\gamma = 1.34 \pm 0.03$, $B = 109 \pm 8$, and $b = 1 \pm 0.1 \times 10^{-3}$. The series was subsequently divided into MS-AS sequences, as in experiments. **(b)** Rescaled Omori-Utsu plot showing the AS rate, $R_{\rm AS}(t|E_{\rm MS})$, following a MS of energy $E_{\rm MS}$, as a function of $(t - t_{\rm MS})/N_{\rm AS}(E_{\rm MS})$, where $N_{\rm AS}(E_{\rm MS})$ is given by Eq. 3 main text. As in Fig. 3d main-text, a perfect collapse is observed for all curves. The different symbols correspond to different values of $E_{\rm MS}$ indicated in the legend. Magenta line is a fit according to Eq. 4 main text with $p = 1.24 \pm 0.03$, $\tau_{\rm min} = 0.24 \pm 0.1$ and $\tau_0 = 7200 \pm 800$.

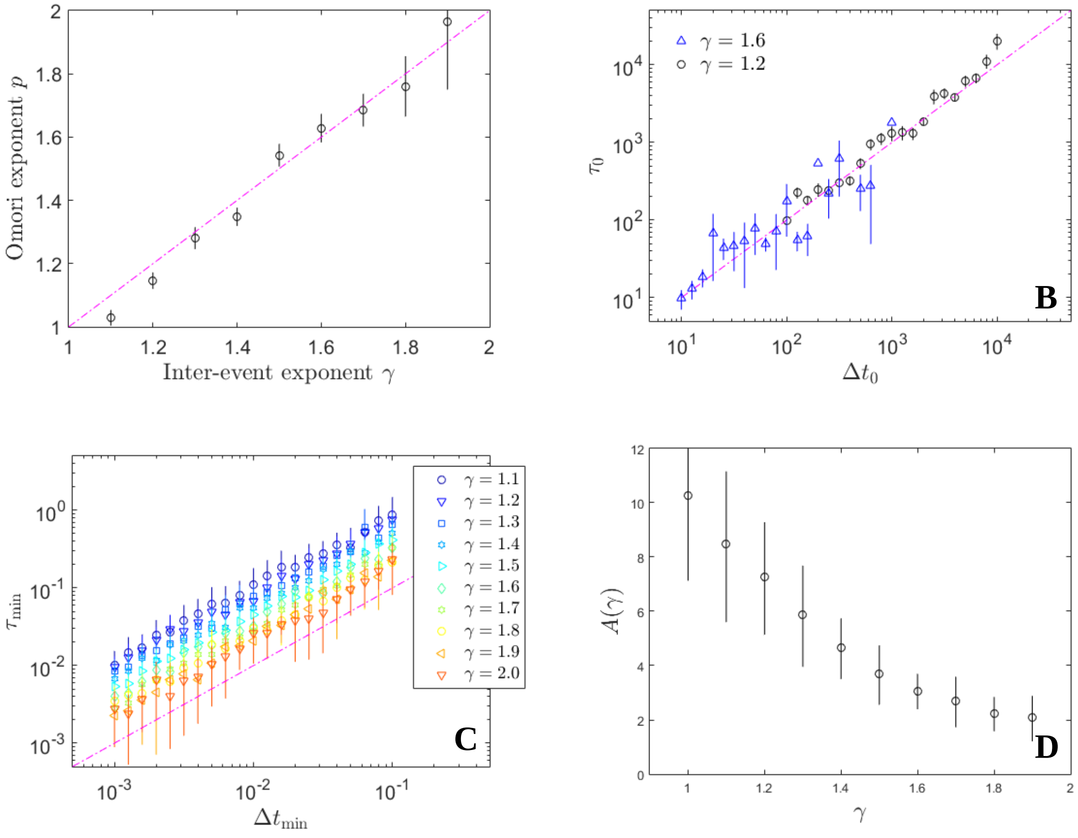

Supplementary Figure 7: **From inter-event distribution to Omori-Utsu law parameters**. Artificial series $\{t, E\}$ were created so that E obeys the Gutenberg-Richter distribution (Main text Eq. 1) with $\{\beta = 0.96, E_{\min} = 10^{-4}, E_0 = 38\}$, and Δt obeys the gamma distribution $P(\Delta t) \propto \Delta t^{-\gamma} \exp(-\Delta t/\Delta t_0)$ for $\Delta t \geq \Delta t_{\min}$ with tunable values for $\{\gamma, \Delta t_{\min}, \Delta t_0\}$. MS-AS sequences were identified in the so-obtained artificial series, the AS rate curves $R_{\text{AS}}(t|E_{\text{MS}})$ were computed and fitted according to the scaled Omori-Utsu law given by Eq. 4 main text (see Supplementary Fig. 6 and associated caption). **(a)** Variation of the fitted Omori-Utsu exponent p as a function of imposed γ for fixed values $\Delta t_{\min} = 10^{-3}$ and $\Delta t_0 = 10^4$. Magenta dash line indicates $p = \gamma$. **(b)** Variation of the fitted Omori-Utsu upper time scale τ_0 as a function of imposed Δt_0 for a fixed value $\Delta t_{\min} = 10^{-3}$ and two different values γ. For each individual fit of $R_{\text{AS}}(t|E_{\text{MS}})$ with Eq. 4 main text, the p exponent was kept fixed to $p = \gamma$. The axes are logarithmic. Magenta dash line indicates $\tau_0 = \Delta t_0$. **(c)** Variation of the fitted Omori-Utsu lower time scale $\tau_{\min}$ as a function of imposed $\Delta t_{\min}$ for a fixed value $\Delta t_{\min} = 10^{-3}$ and different values γ. For each individual fit, p and τ_0 were imposed to $p = \gamma$ and $\tau_0 = \Delta t_0$, respectively. The axes are logarithmic. Magenta dash line indicates $\tau_{\min} = \Delta t_{\min}$. $\tau_{\min}$ is found to be proportional to $\Delta t_{\min}$, with a prefactor $A(\gamma)$ decreasing with γ. **(d)** Variation of this prefactor as a function of γ. In all panels, errorbars indicate a 95% confident interval.

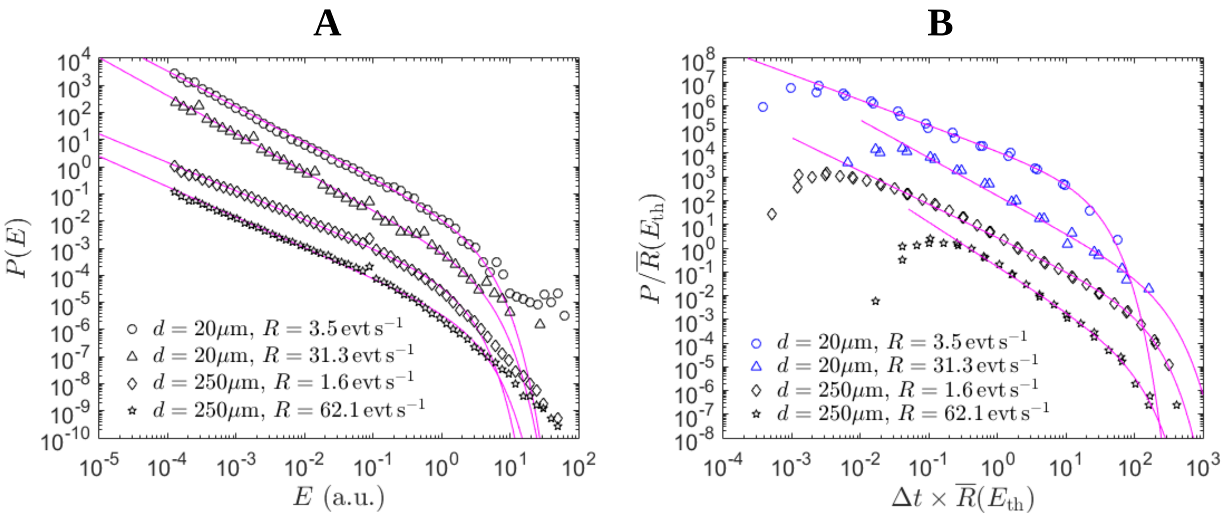

Supplementary Figure 8: **Effect of the solid microstructure on the energy and inter-event time distributions**. **(a)** Distribution of AE energy in fracture experiments driven in two specimens with different microstructure length-scales, d. For each material, two driving rates were used, yielding two different values for the activity rate R. The values $\{d, R\}$ are indicated in the legend. Magenta lines are gamma fit $P(E) \propto E^{-\beta} \exp(-E/E_0)$ for $E \geq E_{\rm min} = 10^{-4}$. The obtained fitted values are: $\{\beta = 1.12 \pm 0.03, E_0 = 1.89 \pm 0.12\}$ for $\{d = 233\ \mu\text{m}, R = 62.1\ \text{AE.s}^{-1}\}$, $\{\beta = 1.05 \pm 0.03, E_0 = 1.05 \pm 0.12\}$ for $\{d = 233\ \mu\text{m}, R = 1.6\ \text{AE.s}^{-1}\}$, $\{\beta = 1.41 \pm 0.03, E_0 = 2.12 \pm 0.75\}$ for $\{d = 24\ \mu\text{m}, R = 31.3\ \text{AE.s}^{-1}\}$, $\{\beta = 1.32 \pm 0.02, E_0 = 1.85 \pm 0.22\}$ for $\{d = 24\ \mu\text{m}, R = 3.5\ \text{AE.s}^{-1}\}$. **(b)** Scaled distribution of the recurrence time Δt for AE with $E > E_{\rm th}$ in the same fracture experiments. As in Fig. 2c in main text, Δt has been rescaled by the mean activity rate $R(E_{\rm th})$ for AE with $E > E_{\rm th}$. For each experiments, two values have been selected for $E_{\rm th}$: $E_{\rm th} = 10^{-2}$ and $E_{\rm th} = 10^{-1}$. Magenta curves are gamma function $f(x) \propto x^{-\gamma} \exp(-x/B)$. The obtained fitted values are: $\{\gamma = 1.87 \pm 0.11, B = 46 \pm 9\}$ for $\{d = 233\ \mu\text{m}, R = 62.1\ \text{AE.s}^{-1}\}$, $\{\gamma = 1.40 \pm 0.04, B = 71 \pm 11\}$ for $\{d = 233\ \mu\text{m}, R = 1.6\ \text{AE.s}^{-1}\}$, $\{\gamma = 1.59 \pm 0.11, B = 120 \pm 99\}$ for $\{d = 24\ \mu\text{m}, R = 31.3\ \text{AE.s}^{-1}\}$, $\{\gamma = 1.07 \pm 0.05, B = 11 \pm 2\}$ for $\{d = 24\ \mu\text{m}, R = 3.5\ \text{AE.s}^{-1}\}$. $\pm$ stands for 95% confident interval and in both panels, axes are logarithmic. In both panels **(a)** and **(b)**, the curves associated to the different sets of $\{d, R\}$ were offset along the y axis.

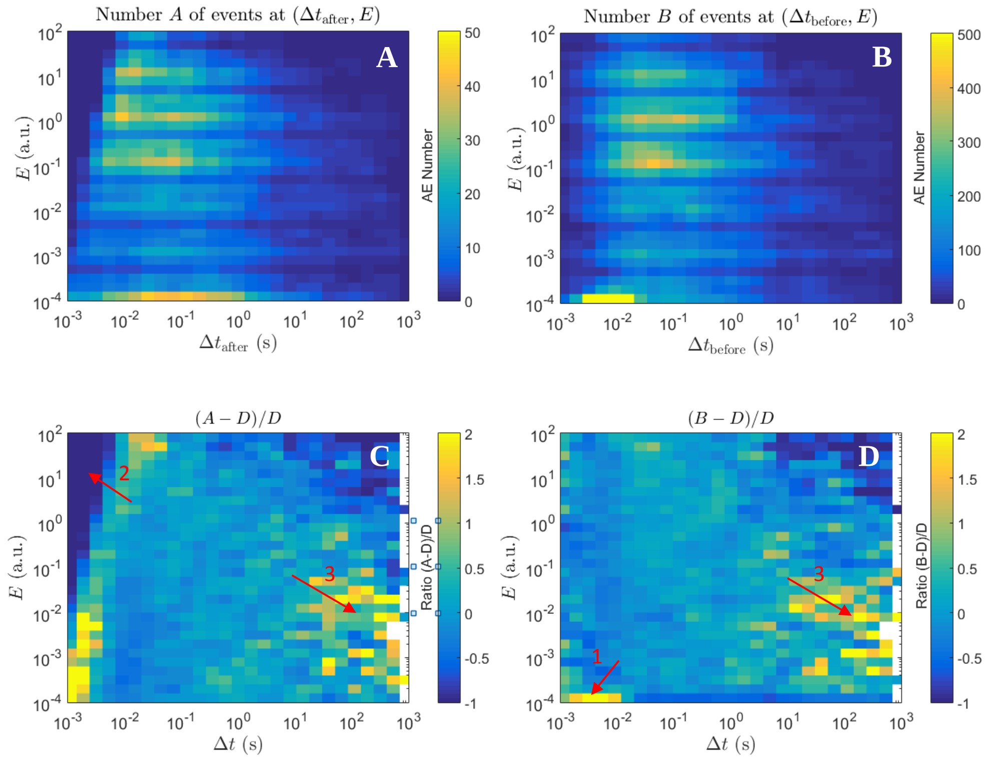

Supplementary Figure 9: **Temporal correlations in the AE series** were characterized using the procedure proposed in Ref.[4]: (i) a triplet $\{E, \Delta t_{\rm before}, \Delta t_{\rm after}\}$ is associated to each event, so that E is the energy of the considered event, $\Delta t_{\rm before}$ is the waiting time preceding it, and $\Delta t_{\rm after}$ is that following it. (ii) 2D maps of the numbers B and A of events falling into boxes $(\Delta t_{\rm before}, E)$ and $(\Delta t_{\rm after}, E)$ are computed; they are plotted in panels **a** and **b**, respectively. (iii) These so-obtained maps are compared with the map, referred to as D, that would have been obtained in a situation where waiting times and energies are uncorrelated (obtained by redistributing randomly the values of the AE energies while keeping their initial occurrence time). The relative differences $(A-D)/D$ and $(B-D)/D$ are plotted in panels **c** and **d**, respectively. In all panels, the axis are logarithmic. The three red arrows in panels **c** and **d** highlight the correlations: (1) When preceding events are considered, events are observed to concentrate around $(\Delta t_{\rm before}, E) = (\tau_{\rm min} \simeq 0.06 \text{ s}, E_{\rm min} \simeq 10^{-4})$ where $E_{\rm min}$ is the lower cutoff for energy (sensitivity of the system) and $\tau_{\rm min}$ is the characteristic time intervening in the Omori law. This reflects the fact that $E_{\rm min}$ and $\tau_{\rm min}$ are the most likely behavior for E and Δt (position of the maximum in the Figs. 2(a) and 2(b) of the main text): (2) When following events are considered, there exists a gap at high E/low $\Delta t_{\rm after}$, or, to be more precise, a gap for $\Delta t_{\rm after} < \Delta t^{c}_{\rm after} \sim E^{a}$ with $a \approx 1/6$. This might be a signature of the short-time aftershock incompleteness (STAI) documented in seismology (see main text): Some events right after a high-energy event are missing because their waveform has been drown in that of their predecessor. (3) For both preceding and following events, a (slightly) larger density for small energy and large waiting times is observed. This indicates the existence of "inactivity" times characterized by long waiting times and low energy events, as in Ref.[4]. In this experiment, the microstructure length-scale is $d = 583\,\mu$m and the crack speed $\overline{v} = 2.7\,\mu\text{m}\,\text{s}^{-1}$.

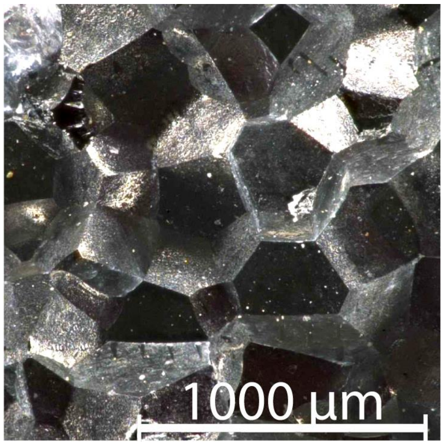

Supplementary Figure 10: **Microscope image of the post-mortem fracture surfaces** corresponding to the experiments analyzed in Figs. 2 and 3 in the main text. Note the facet-like structure illustrating the intergranular fracture mode and the absence of visible porosity.

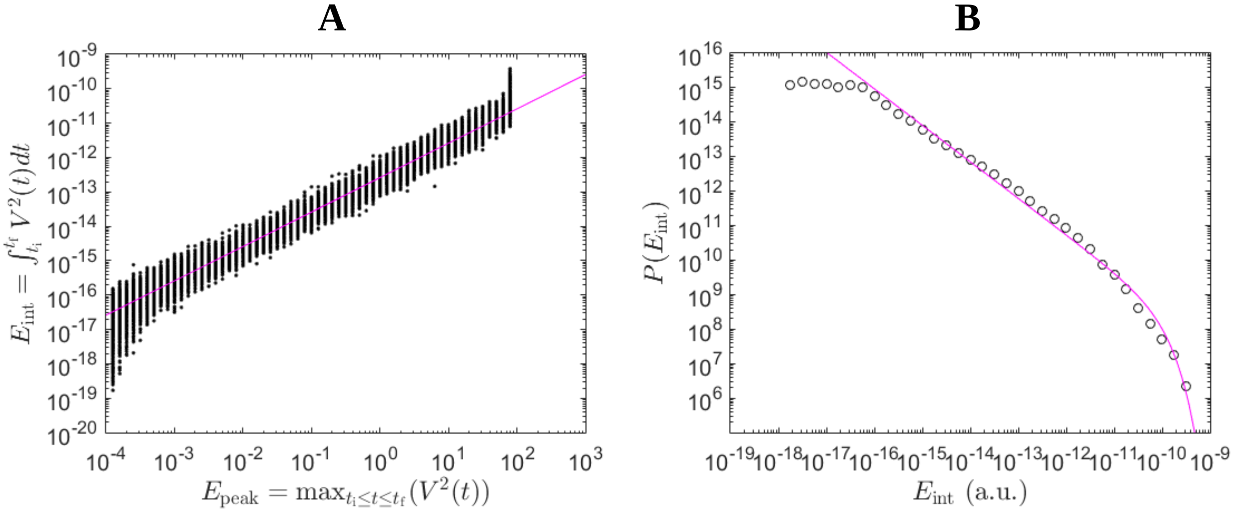

Supplementary Figure 11: **Effect of the definition for AE energy**. There exists several ways to define the energy, E, of an AE. The two most widely used are: (i) $E = E_{\mathrm{peak}}$ defined as the squared maximum value of the signal $V(t)$ recorded by the transducer during the considered event; (ii) $E = E_{\mathrm{int}}$ defined as the integral of $V^2(t)$ over the event duration. **(a)** Plot of E_{int} as a function of E_{peak} for all the AE of the experiment used in Fig. 2 main text (micro-structure length-scale: $d = 583\,\mu\mathrm{m}$, crack speed $\overline{v} = 2.7\,\mu\mathrm{m\,s^{-1}}$). The axis are logarithmic and the straight magenta line denotes proportionality: $E_{\mathrm{int}} = CE_{\mathrm{peak}}$ with $C = 2.6 \times 10^{-13}$. **(b)** Distribution of AE energy in the same experiment when $E = E_{\mathrm{int}}$. The axis are logarithmic and the solid magenta line is a gamma function $P(E_{\mathrm{int}}) \propto E_{\mathrm{int}}^{-\beta^*} \exp(-E_{\mathrm{int}}/E_0^*)$ for $E_{\mathrm{int}} \geq E_{\mathrm{min}}^* = 5 \times 10^{-17}$, with fitted parameters $\beta^* = 1.04 \pm 0.06$ and $E_0^* = 7 \pm 2 \times 10^{-11}$. In this experiment, the microstructure length-scale is $d = 583\,\mu\mathrm{m}$ and the crack speed $\overline{v} = 2.7\,\mu\mathrm{m\,s^{-1}}$.

Supplementary Note 1: Interrelation between the parameters at play in the scaled distribution of inter-event times

Calling Δt the time separating two successive AE events of energy larger than a prescribed threshold E_{th}, and $R(E_{\mathrm{th}})$ the average rate of such events, the scaled recurrence times $u = \Delta t \times R(E_{\mathrm{th}})$ has been observed to obey a universal gamma function (Main text Eq. 2):

$$P(u) = Cu^{-\gamma} \exp(-u/B) \quad \text{for} \quad u > b \tag{1}$$

The exponent γ and the two time scales b and B intervening in this equation can be related. First, it should be noted that $\int_b^\infty P(u)\mathrm{d}u = 1$, hence $\int_b^\infty u^{-\gamma}\exp(-u/B)\mathrm{d}u = 1/C$. The variable change $u \to v = u/B$ in the integral term then leads to:

$$1/C = B^{1-\gamma}\Gamma(1-\gamma, b/B), \tag{2}$$

where $\Gamma(s,x) = \int_x^\infty t^{s-1}e^{-t}\mathrm{d}t$ is the upper incomplete gamma function. Using the recurrence properties of this function, one gets:

$$1/C = B^{1-\gamma}\left(\frac{1}{1-\gamma}\Gamma(2-\gamma, b/B) - \frac{1}{1-\gamma}\left(\frac{b}{B}\right)^{1-\gamma} e^{-b/B}\right) \tag{3}$$

Assuming $\gamma > 1$ and $b \ll B$, Supplementary Eq. 3 reduces to:

$$C \approx \frac{(\gamma - 1)b^{\gamma-1}}{1 - \Gamma(2-\gamma)(b/B)^{\gamma-1}} \tag{4}$$

where $\Gamma(x)$ is the gamma function. We choose here to go to the first order in $(b/B)^{\gamma-1}$ rather than restricting the calculation to the leading term, $C \approx (\gamma - 1)b^{\gamma-1}$, since the exponent γ can be close to 1.

Second, it should be noted that the mean value $< u >$ of the scaled recurrence time is equal to unity. In other words, $\int_b^\infty uP(u)\mathrm{d}u = 1$, hence $C\int_b^\infty u^{1-\gamma}\exp(-u/B)\mathrm{d}u = 1$. The variable change $u \to v = u/B$ in the integral term leads to $CB^{2-\gamma}\Gamma(2-\gamma, b/B) = 1$. The assumption that $b \ll B$ leads to:

$$CB^{2-\gamma}\Gamma(2-\gamma) \approx 1 \tag{5}$$

Finally, by combining Supplementary Eqs. 4 and 5, the following relation is obtained:

$$(\gamma - 1)\Gamma(2-\gamma)b^{\gamma-1}B^{2-\gamma} \approx 1 - \Gamma(2-\gamma)(b/B)^{\gamma-1} \tag{6}$$

Supplementary Note 2: Relation between the energy distribution and the productivity law for AS

The lack of correlation between occurrence time and energy permits to infer the productivity law $N_{\mathrm{AS}}\, vs.\, E_{\mathrm{MS}}$ (mean number of triggered AS as a function of the mainshock (MS) energy) from the distribution of energy, $P(E)$.

The total number of events with an energy smaller that the prescribed energy E_{MS} for MS gives the total number of AS in the catalog (summed over all AS sequences), $\mathcal{N}^{\mathrm{AS}}_{\mathrm{total}}$. The total number of events with an energy larger than E_{MS} gives, by definition, the total number of MS, and hence the total number of AS sequences, $\mathcal{N}^{\mathrm{sequences}}_{\mathrm{total}}$. The mean number of AS per sequence $N_{\mathrm{AS}}(E_{\mathrm{MS}})$ is then given by the ratio between the two. Calling N_{total} the total number of AE in the considered catalog and $F(E) = \int_{E_{\min}}^{E} P(E)\mathrm{d}E$ the cumulative distribution of energy, $\mathcal{N}^{\mathrm{AS}}_{\mathrm{total}}$ and $\mathcal{N}^{\mathrm{sequences}}_{\mathrm{total}}$ write $\mathcal{N}^{\mathrm{AS}}_{\mathrm{total}} = N_{\mathrm{total}} \times F(E_{\mathrm{MS}})$ and $\mathcal{N}^{\mathrm{sequences}}_{\mathrm{total}} = N_{\mathrm{total}} \times (1 - F(E_{\mathrm{MS}}))$, respectively. As a result, $N_{\mathrm{AS}}(E_{\mathrm{MS}}) = \mathcal{N}^{\mathrm{AS}}_{\mathrm{total}} / \mathcal{N}^{\mathrm{sequences}}_{\mathrm{total}}$ writes:

$$N_{\mathrm{AS}}(E_{\mathrm{MS}}) = \frac{F(E_{\mathrm{MS}})}{1 - F(E_{\mathrm{MS}})} \tag{7}$$

When $P(E)$ takes a simple Gutenberg-Richter power-law form, from $E_{\min}$ to ∞, with an exponent $\beta > 1$, $F(E_{\mathrm{MS}}) = 1 - (E_{\mathrm{MS}}/E_{\min})^{1-\beta}$ and $1 - F(E_{\mathrm{MS}}) = (E_{\mathrm{MS}}/E_{\min})^{1-\beta}$. Assuming that $E_{\mathrm{MS}} \gg E_{\min}$, this leads to:

$$N_{\mathrm{AS}}(E_{\mathrm{MS}}) \approx \left(\frac{E_{\mathrm{MS}}}{E_{\min}}\right)^{\alpha} \quad \text{with} \quad \alpha = \beta - 1 \tag{8}$$

Supplementary Note 3: Relation between the energy distribution and Båth's law

As above, the lack of correlation between occurrence time and energy permits to infer Båth's law $\Delta M \; vs. \, E_{\mathrm{MS}}$ (mean difference in magnitude between the MS and its largest AS, as a function of the MS energy) from the distribution of energy, $P(E)$. Let us call $F_{\mathrm{ASmax}}(E|N_{\mathrm{AS}})$ the probability that the largest AS of a sequence of size N_{AS} is smaller than E. All the other AS of the sequence have an energy smaller than E:

$$F_{\mathrm{ASmax}}(E|N_{\mathrm{AS}}) = F(E)^{N_{\mathrm{AS}}} \tag{9}$$

The mean value $< \max(E_{\mathrm{AS}}|E_{\mathrm{MS}}) >$ of the energy of the largest event over the sequences triggered by a MS of energy E_{MS} then writes:

$$< \max(E_{\mathrm{AS}}|E_{\mathrm{MS}}) >= \int_{E_{\min}}^{E_{\mathrm{MS}}} E \frac{\mathrm{d}}{\mathrm{d}E} F_{\mathrm{ASmax}}(E|N_{\mathrm{AS}}) \mathrm{d}E = N_{\mathrm{AS}}(E_{\mathrm{MS}}) \int_{E_{\min}}^{E_{\mathrm{MS}}} E F(E)^{N_{\mathrm{AS}}-1} P(E) \mathrm{d}E, \tag{10}$$

where $N_{\mathrm{AS}}(E_{\mathrm{MS}})$ is given by Supplementary Eq. 7. The difference in magnitude $\Delta M(E_{\mathrm{MS}}) = \log(< \max(E_{\mathrm{AS}}|E_{\mathrm{MS}}) > / E_{\mathrm{MS}})$ can then be deduced. As for the productivity law, $\Delta M(E_{\mathrm{MS}})$

takes a simpler form when $P(E)$ takes a simple power-law form $P(E) \propto E^{-\beta}$. Indeed, Supplementary Eq. 10 writes:

$$< \max(E_{\mathrm{AS}}|E_{\mathrm{MS}}) >= (\beta-1)N_{\mathrm{AS}}(E_{\mathrm{MS}})E_{\mathrm{min}} \int_1^{E_{\mathrm{MS}}/E_{\mathrm{min}}} u^{1-\beta}(1-u^{1-\beta})^{N_{\mathrm{AS}}(E_{\mathrm{MS}})-1}\mathrm{d}u, \quad (11)$$

with $N_{\mathrm{AS}}(E_{\mathrm{MS}})$ given by Supplementary Eq. 8. This yields:

$$\Delta M(x|\beta) = -\log_{10}\left((\beta-1)x^{\beta-2}\int_1^x u^{1-\beta}(1-u^{1-\beta})^{u^{\beta-1}-1}\mathrm{d}u\right) \quad (12)$$

where $x = E_{\mathrm{MS}}/E_{\mathrm{min}}$. Numerical computation shows that, as x goes to infinite, $\Delta M(x|\beta)$ converges toward a finite value $\Delta M_\infty(\beta)$ (Supplementary Fig. 4a), the value of which is plotted on Supplementary Fig. 4b.

It is finally interesting to note that, after a short transient and before reaching the plateau value ΔM_∞, $\Delta M(x)$ predicted by Supplementary Eq. 12 increases with the MS energy, as observed in seismology. Conversely, in the experiments reported here, ΔM decreases with E_{MS} (main text Fig. 3B). This decrease comes for the specific form of $P(E)$ in our experiments, with the presence of an exponential cutoff made all the more important so as $\beta \approx 1$ (even smaller), which sets all the moments of $P(E)$.

Supplementary Note 4: Interrelation between the parameters at play in the scaled Omori-Utsu law for AS

The mean number of triggered AS, $N_{\mathrm{AS}}(E_{\mathrm{MS}})$, writes $N_{\mathrm{AS}} = \int_{t_{\mathrm{MS}}}^{\infty} R_{\mathrm{AS}}(t|E_{\mathrm{MS}})\mathrm{d}t$ where the rate of AS events following a MS of energy E_{MS}, $R_{\mathrm{AS}}(t|E_{\mathrm{MS}})$, follows the scaled Omori-Utsu law (Main text Eq. 4). This yields:

$$N_{\mathrm{AS}}(E_{\mathrm{MS}}) = \int_{t_{\mathrm{MS}}}^{\infty} \frac{R_0}{\left(1 + \frac{t - t_{\mathrm{MS}}}{\tau_{\mathrm{min}} N_{\mathrm{AS}}(E_{\mathrm{MS}})}\right)^p} \exp\left(-\frac{t - t_{\mathrm{MS}}}{\tau_0 N_{\mathrm{AS}}(E_{\mathrm{MS}})}\right) \mathrm{d}t \tag{13}$$

A first variable change $t \to u = (t - t_{\mathrm{MS}})/N_{\mathrm{AS}}(E_{\mathrm{MS}})$ in the right-handed integral yields:

$$1 = R_0 \int_0^{\infty} \frac{1}{(1 + u/\tau_{\mathrm{min}})^p} e^{-u/\tau_0} \mathrm{d}u \tag{14}$$

A second variable change $u \to v = u + \tau_{\mathrm{min}}$ yields:

$$1 = R_0 \tau_{\mathrm{min}}^p e^{\tau_{\mathrm{min}}/\tau_0} \int_{\tau_{\mathrm{min}}}^{\infty} v^{-p} e^{-v/\tau_0} \mathrm{d}v \tag{15}$$

A third variable change $v \to w = v/\tau_0$ yields:

$$1 = R_0 \tau_{\mathrm{min}}^p \tau_0^{1-p} e^{\tau_{\mathrm{min}}/\tau_0} \Gamma(1 - p, \tau_{\mathrm{min}}/\tau_0) \tag{16}$$

Finally, providing $p > 1$ and assuming $\tau_{\min}/\tau_0 \ll 1$, the use of the recurrence properties on the incomplete gamma function yields:

$$R_0 \approx \frac{(p-1)/\tau_{\min}}{1-(\tau_{\min}/\tau_0)^{p-1}} \tag{17}$$

Here again, we did not restrict the calculation to the leading order, $R_0 \approx (p-1)/\tau_{\min}$, but chose to go to first order in $(\tau_{\min}/\tau_0)^{p-1}$ since the exponent p can be close to 1.

Supplementary Note 5: Relation between the inter-event distribution and Omori-Utsu law

Artificial series of AE events $\{t_i, E_i\}$ were created using the following procedure: i) The inter-event times $\Delta t_i = t_i - t_{i+1}$ were pulled randomly according to the gamma distribution $P(\Delta t) \propto \Delta t^{-\gamma} \exp(-\Delta t/\Delta t_0)$ for $\Delta t \geq \Delta t_{\min}$; ii) the occurrence times were defined as $t_i = \sum_{k=1}^{i} \Delta t_k$ ($t_0 = 0$); and iii) The event energies E_i were pulled randomly according to the Gutenberg-Richter distribution given by Eq. 1 in main text. In the so-obtained series, the distribution of scaled recurrence time $R(E_{\text{th}}) \times \Delta t$ obeys Eq. 2 – main-text (Supplementary Fig. 6a), illustrating the self-similarity of the series. It is recalled here that $R(E_{\text{th}})$ denotes the mean activity rate for events with an energy $E > E_{\text{th}}$. MS-AS sequences were identified and analyzed as in experiments. In particular, the time evolution of mean AS rate, $R_{\text{AS}}(t|E_{EM})$, following a MS of energy E_{MS} was computed. As shown in Supplementary Fig. 6b, it follows the very same scaled Omori-Utsu law (Eq. 4 – main-text) as in experiments.

These artificial series were used to unravel the relations between the set of parameters $\{\gamma, \Delta t_{\min}, \Delta t_0\}$ characterizing the inter-event distribution and the parameters $\{p, \tau_{\min}, \tau_0\}$ characterizing the scaled Omori-Utsu law. The parameters associated to the Gutenberg-Richter law for energy were set to $\beta = 0.96$, $E_{\min} = 10^{-4}$ and $E_0 = 38$ to coincide with those measured in the artificial rock (microstructure length-scale $d = 580\ \mu\mathrm{m}$) experimentally investigated. Supplementary Fig. 7a presents the variations of the Omori-Utsu exponent p as a function of the exponent γ at fixed values $\Delta t_{\min}$ and Δt_0. $p \approx \gamma$ and is independent of both $\Delta t_{\min}$ and Δt_0. Conversely, the latter set the value of Omori upper time scale: $\tau_0 \approx \Delta t_0$ (Supplementary Fig. 7b) and is independent of both $\Delta t_{\min}$ and γ. Finally, $\tau_{\min}$ is found to depend on both $\Delta t_{\min}$ and γ: $\tau_{\min} \approx A(\gamma)\Delta t_{\min}$ (Supplementary Fig. 7c) where the prefactor $A(\gamma)$ is a decreasing function of γ, provided in Supplementary Fig. 7d. These relations can be rewritten as a function of the dimensionless parameters b and B rather than $\Delta t_{\min}$ and Δt_0:

$$p \approx \gamma, \quad , \tau_{\min} \approx A(\gamma)\Delta t_{\min} = A(\gamma) b/R, \quad \tau_0 \approx \Delta t_0 = B/R, \tag{18}$$

where R is recalled to be the activity rate.

Supplementary Note 6: Predicted relation between upper Omori-Utsu time scale and mean crack speed

The mean activity rate R is given by:

$$R \approx \overline{v}H/d^2 \tag{19}$$

where $\overline{v}$, H and d denote the mean crack speed, the specimen thickness and the microstructure length-scale, respectively (main text).

Let us now compute the mean inter-event time, $\langle \Delta t \rangle$. First, it is to recall that $P(\Delta t)$ follows a gamma distribution $P(\Delta t) = C\Delta t^{-\gamma} \exp(-\Delta t/\tau_0)$ where the constant C is set by the normalization $\int_{\Delta t_{\min}}^{\infty} P(\Delta t)\mathrm{d}\Delta t = 1$: Assuming $\Delta t_{\min} \ll \Delta t_0$, to leading order, $C \approx (\gamma - 1)\Delta t_{\min}^{\gamma-1}$. Second, it should be noted that $\langle \Delta t \rangle = \int_{\Delta t_{\min}}^{\infty} \Delta t P(\Delta t)\mathrm{d}\Delta t$, hence $\langle \Delta t \rangle = C \int_{\Delta t_{\min}}^{\infty} \Delta t^{1-\gamma} \exp(-\Delta t/\tau_0)\mathrm{d}\Delta t$. The assumption $\Delta t_{\min} \ll \Delta t_0$ and the knowledge of the value of C leads to:

$$\langle \Delta t \rangle \approx \Gamma(2-\gamma)(\gamma-1)\Delta t_{\min}^{\gamma-1}\Delta t_0^{2-\gamma} \tag{20}$$

For sake of simplicity, the expression here has been reduced to the leading order, and not to the first order in $(b/B)^{\gamma-1}$ as for Supplementary Eq. 6. As $R = 1/\langle \Delta t \rangle$, the combination of Supplementary Eqs. 19 and 20 yields:

$$\frac{d^2}{\overline{v}H} \approx \Gamma(2-\gamma)(\gamma-1)\Delta t_{\min}^{\gamma-1}\Delta t_0^{2-\gamma}, \tag{21}$$

and by using the relations between the inter-event and Omori parameters (Supplementary Eq. 18), one gets:

$$\tau_0 \approx \left(\Gamma(2-p)(p-1)A(p)^{p-1}\right)^{-1/(2-p)} \tau_{\min} \left(\frac{d^2}{H\tau_{\min}\overline{v}}\right)^{1/(2-p)} \tag{22}$$

By assuming p constant and neglecting its dependency with $\overline{v}$, Supplementary Eq. 22 reduces to:

$$\tau_0 \propto 1/\overline{v}^{1/(2-p)} \tag{23}$$

Supplementary References

1. Barés, J., Hattali, M. L., Dalmas, D. & Bonamy, D. Fluctuations of global energy release and crackling in nominally brittle heterogeneous fracture. *Physical Review Letters* **113**, 264301 (2014).

2. van Stiphout, T., Zhuang, J. & Marsan, D. Seismicity declustering, community online resource for statistical seismicity analysis (corssa), doi: 10.5078/corssa-52382934 (2012).

3. Hainzl, S., Scherbaum, F. & Beauval, C. Estimating background activity based on interevent-time distribution. *Bulletin of the Seismological Society of America* **96**, 313–320 (2006).

4. Stojanova, M., Santucci, S., Vanel, L. & Ramos, O. High frequency monitoring reveals aftershocks in subcritical crack growth. *Physical Review Letters* **112**, 115502 (2014).
