# Supplementary material for: Aftershock sequences and seismic-like organization of acoustic events produced by a single propagating crack
Source: Nat Commun. 2018 Mar 28;9:1253. doi: 10.1038/s41467-018-03559-4 (PMC5871842; doi:10.1038/s41467-018-03559-4)
Supplement: Supplementary file 3 — Description of Additional Supplementary Files [file 41467_2018_3559_MOESM3_ESM.pdf]

Description of Supplementary Files

File Name: Supplementary Movie 1
Description: Acoustic imaging of crack propagation in a typical artificial rock. Top: Spatial 3D localization of the AE throughout the sample as time evolves. Blue points show all the events since the beginning, while red points only concern the events having occurred over the last second. Bottom: Crack propagation as observed by means of a camera at the specimen surface. The two movies are synchronized. The size of observation is 130×38×15 mm^3 at top, and 130×38mm^2 at bottom. For this specific experiment, the bead diameter prior sintering was d=583 µm (Methods) and the wedge speed was 16 nm s^{-1}. The film is accelerated and the time is given in between the two movies (in hour). The total duration of the recorded process is about 5h30, yielding a mean crack speed $\bar{v} = 2.7$ µm s^{-1}.
